# Metagenomics Reveals Dominant Unusual Sulfur Oxidizers Inhabiting Active Hydrothermal Chimneys From the Southwest Indian Ridge

**DOI:** 10.3389/fmicb.2022.861795

**Published:** 2022-05-25

**Authors:** Yong Wang, Hong-Yu Bi, Hua-Guan Chen, Peng-Fei Zheng, Ying-Li Zhou, Jiang-Tao Li

**Affiliations:** ^1^Institute for Marine Engineering, Shenzhen International Graduate School, Tsinghua University, Shenzhen, China; ^2^Institute of Deep Sea Science and Engineering, Chinese Academy of Sciences, Sanya, China; ^3^College of Marine Sciences, University of Chinese Academy of Sciences, Beijing, China; ^4^State Key Laboratory of Marine Geology, Tongji University, Shanghai, China

**Keywords:** deep-sea hydrothermal vents, SWIR, thiohalomonadales, metagenomics, red light photosynthesis

## Abstract

The deep-sea hydrothermal vents (DSHVs) in the Southwest Indian Ridge (SWIR) are formed by specific geological settings. However, the community structure and ecological function of the microbial inhabitants on the sulfide chimneys of active hydrothermal vents remain largely unknown. In this study, our analyses of 16S rRNA gene amplicons and 16S rRNA metagenomic reads showed the dominance of sulfur-oxidizing Ectothiorhodospiraceae, *Thiomicrorhabdus*, *Sulfurimonas*, and *Sulfurovum* on the wall of two active hydrothermal chimneys. Compared with the inactive hydrothermal sediments of SWIR, the active hydrothermal chimneys lacked sulfur-reducing bacteria. The metabolic potentials of the retrieved 82 metagenome-assembled genomes (MAGs) suggest that sulfur oxidation might be conducted by Thiohalomonadales (classified as Ectothiorhodospiraceae based on 16S rRNA gene amplicons), Sulfurovaceae, Hyphomicrobiaceae, Thiotrichaceae, Thiomicrospiraceae, and Rhodobacteraceae. For CO_2_ fixation, the Calvin-Benson-Bassham and reductive TCA pathways were employed by these bacteria. In Thiohalomonadales MAGs, we revealed putative phytochrome, carotenoid precursor, and squalene synthesis pathways, indicating a possible capacity of Thiohalomonadales in adaptation to dynamics redox conditions and the utilization of red light from the hot hydrothermal chimneys for photolithotrophic growth. This study, therefore, reveals unique microbiomes and their genomic features in the active hydrothermal chimneys of SWIR, which casts light on ecosystem establishment and development in hydrothermal fields and the deep biosphere.

## Introduction

Deep-sea hydrothermal vents (DSHVs) are located in tectonically active areas where plate boundaries move at different speeds along mid-ocean ridges. DSHV is an important conduit for the exchange of energy and materials between the Earth’s interior and the ocean. Since the first report in 1977, DSHVs as a deep-sea extreme environment have attracted great concerns about the microbial extremophilic inhabitants with respect to early life form, chemoautotrophy, and adaptation (Corliss et al., 1979; Francheteau et al., 1979; Jannasch and Mottl, 1985; Kelley et al., 2005; Dick, 2019). The mixture of cold, oxic deep-sea water, and highly reducing fluids with high concentrations of hydrogen, sulfide, and methane was ideal for chemoautotrophs and C1 oxidizers (Dick et al., 2013). The complex dynamic habitats have steep thermal and chemical gradients, and different microbial populations are simultaneously involved in many important biogeochemical processes, such as the nitrogen, sulfur, and carbon cycles that also occur in symbionts of megafauna around DSHV (Jannasch and Mottl, 1985; Connelly et al., 2012). Strong dynamics of geochemistry and temperature provide a wide range of habitats for deep-sea microorganisms, and therefore, niche specificity of microbes has been demonstrated in different environmental settings by previous studies (Dick, 2019 and references therein).

Sulfide samples from inactive and active hydrothermal vents are distinct in microbial community structure and ecological function in the Indian Ocean (Zhang et al., 2016; Han et al., 2018; Adam et al., 2020; Hou et al., 2020). Sulfur oxidizing bacteria (SOB) dominate various hydrothermal sediments and flumes and are classified to be aerobes (e.g., SUP05 and *Beggiatoa* from Gammaproteobacteria), microaerobes (e.g., *Sulfurimonas* and *Sulfurovum* from Epsilonbacteraeota), and anaerobes (e.g., *Caminibacter* and *Nautila* from Epsilonbacteraeota) (López-García et al., 2003; Dick, 2019; Meier et al., 2019; Hou et al., 2020) with preference to different electron donors. For the microaerobic SOB, the *cbb3*-type cytochrome c oxidase was involved in adaptation to low oxygen concentrations for respiration (Hou et al., 2020; Dong et al., 2021); however, the mechanism for reducing the damage by high oxygen flux is largely unknown. Considering the highly variable microenvironments adjacent to an active hydrothermal vent, there are perhaps much more SOB species that have evolved to adapt to the varying temperature and redox conditions. A photoautotrophic bacterium has been isolated from a hydrothermal chimney (Beatty et al., 2005). Evidence shows that this bacterial isolate from the *Prosthecochloris* genus can absorb weak ultra-red light as energy to fix CO_2_ and oxidize H_2_S. The abundance and distribution of such photoautotrophic SOB in the dark ocean are still unclear up to date.

Since the first report in the Southwest Indian Ridge (SWIR) more than a decade ago (German et al., 1998), multiple hydrothermal fields have been discovered in the Indian Ocean (Van Dover et al., 2001). Low-temperature hydrothermal activity because of the effect of an ultra-slow spreading speed of tectonic plates was reported in the SWIR (Tao et al., 2012). The discovery of hydrothermal fields in such an ultra-slow spreading ridge provides an unprecedented opportunity to understand microbially mediated biogeochemistry. A serpentinite-hosted hydrothermal site and distinct microbial community resembling those of the Lost City hydrothermal field in the Atlantic Ocean were reported in a magma-poor area of the eastern SWIR (Lecoeuvre et al., 2021). The relatively low temperature and serpentinization of SWIR hydrothermal fields (Zhou and Dick, 2013) had probably shaped the microbial inhabitants in the hydrothermal fields. However, microbial genomics studies on the SWIR hydrothermal areas are still limited. *Thiomicrorhabdus* has been reported as a dominant SOB in SWIR (Cao et al., 2014), and was also isolated from the other hydrothermal vents (Brazelton and Baross, 2010; Scott et al., 2019). A recent study revealed a large number of Gammaproteobacteria with sulfur oxidation potentials using nitrate and oxygen as electron acceptors in the SWIR inactive hydrothermal sediments (Dong et al., 2021). We employed metagenomics to compare the microbiomes in the active hydrothermal vents with those from inactive ones to reveal microbial community structure and ecological function driven by active “black-smoker” hydrothermal seepage in SWIR. We hereby report a unique microbiome exclusively composed of Thiohalomonadales-dominated SOB, which has not been reported in DSHVs and presumably plays a critical role in the initiation of the deep-sea hydrothermal ecosystem in SWIR.

## Materials and Methods

### Sample Collection and Mineral Analysis

**FIGURE 1:**
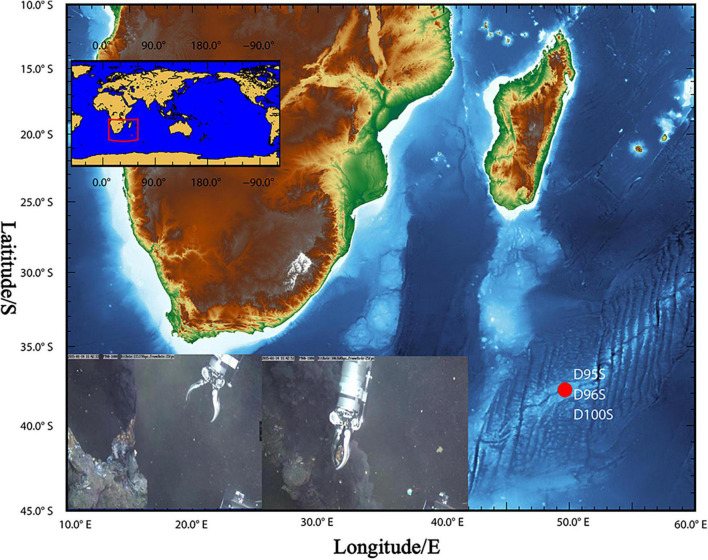
Sampling site at “Longqi” hydrothermal field. During *R/V* DY35, two chimney rocks (D96S and D100S) were obtained by the “Jiaolong” submersible from active sulfide chimney bodies (inset figures) at “Longqi” hydrothermal field in the Southwest Indian Ocean. A sulfide sample (D95A) was also collected from a nearby inactive chimney by the submersible. The details of the samples are listed in Supplementary Table 1.

During the *R/V* DY35 cruise, the manned submersible “Jiaolong” collected sulfide samples from hydrothermal chimneys by dives no. 96 (49.65°E, 37.78°S; depth: 2,768) and no. 100 (49.65°E, 37.78°S; depth: 2,755 m) in SWIR (Figure 1, Supplementary Table 1, and Supplementary Figure 1). A sulfide chimney sample was obtained from an inactive hydrothermal vent (49.63°E, 37.77°S; depth: 2,789 m) by dive no. 95 at a depth of 2,789 m. The samples were stored in sterile bags and maintained at –80°C until use. The temperature and pH of the hydrothermal fluid were measured *in situ* with a Miniature Autonomous Plume Recorder (MAPR) (Baker and Milburn, 1997).

An X-ray diffraction (XRD) analysis was carried out to determine the major minerals of the sulfide chimneys using an Empyrean X-ray diffractometer (PANalytical, Malvern, United Kingdom). The detection parameters were Cu Kα radiation at 45 kV and 40 mA; Goniometer PW3050/60 (Theta/Theta); scanning from 3° to 85° with 0.03 step size (°2Th). The mineral components were converted to wt%.

### High-Throughput Sequencing and Analyses of 16S rRNA Gene Amplicons

Genomic DNA of sulfide samples was extracted from a 2 g sample using the MO BIO Powersoil DNA isolation kit (Qiagen, Carlsbad, CA, United States). The quality and quantity of the DNA extractions were checked by the Qubit 2.0 fluorometer (Invitrogen, Carlsbad, CA, United States). Then, 16S rRNA gene fragments from V3–V4 regions were amplified with a set of universal primers 341F (5′-CCTAYGGGRBGCASCAG-3′) and 802R (5′-TACNVGGGTATCTAATCC-3′) (Wang and Qian, 2009) using 1 ng DNA as a template. PCR conditions of the amplification were performed as follows: initial denaturation at 98°C for 10 s; 28 cycles of denaturation at 98°C for 10 s, annealing at 50°C for 15 s, and extension at 72°C for 30 s; and a final extension at 72°C for 5 min. A PCR replicate was performed. The mixed barcoded 16S rRNA gene amplicons and 100 ng genomic DNA were used for Illumina library preparation using the TruSeq Nano DNA LT kit (Illumina, San Diego, CA, United States), separately, and were sequenced on an Illumina Miseq sequencer to obtain 2 × 300 bp paired-end sequencing reads (Illumina, San Diego, CA, United States).

The sequencing reads of 16S rRNA gene amplicons were checked by FASTQC (v0.11.8)^1^ and merged by the QIIME 1 workflow (Kuczynski et al., 2011). The adaptors and low-quality reads were trimmed by the NGS QC Toolkit (v2.3.3) (Patel and Jain, 2012). Operational taxonomic units (OTUs) at 97% similarity were selected between the qualified reads by the QIIME 1 workflow (Kuczynski et al., 2011). Taxonomic classification of the OTUs was performed with vsearch against the SILVA database v138 (Quast et al., 2013). A principal component analysis (PCoA) was performed to estimate the similarity between the microbial communities at the family level using vegan in the R package (Dixon, 2003).

### Metagenomics Analyses of Microbial Community and Metabolic Potentials

Raw reads of metagenomes were qualified by removing adapters and then filtered by using fastp (version 0.20.0) (Chen et al., 2018) with parameters (-w 16 -q 20 -u 20 -g -c -W 5 -3 -l 50). Low-quality reads (assigned by a quality score < 20 for >20% of the read length), and those shorter than 50 bp and unpaired were removed. Metagenomics reads that were mapped onto sequences in an in-house contaminant database (such as, sequences of human, mouse, and common laboratory contaminant bacterial genomes downloaded from the National Center for Biotechnology Information [NCBI]) (Huang et al., 2019) by Bowtie2 (version.2.4.1) (Langmead and Salzberg, 2012) were discarded.

Furthermore, 16S miTags were extracted from qualified metagenomic reads using rna_hmm3.py (Huang et al., 2019), which employs HMMER (version 3.1b2) to predict ribosomal RNA gene fragments from both forward and reverse metagenomic reads. An in-house python script was used to extract 16S miTags. The 16S miTags were imported into Qiime1 with a setting of –type “SampleData[Sequences]” and dereplicated redundancy to yield representative 16S miTag sequences. The classify-consensus-vsearch command in QIIME v1.9.1 (Kuczynski et al., 2011) was used to classify the representative 16S miTags using the SILVA SSU database v138 as a reference (Quast et al., 2013), as mentioned above.

The qualified reads were assembled using SPAdes (v3.13) (Nurk et al., 2013) with a kmer set of 21, 33, 55, 77, 99, and 127 under the “—careful” mode (metagenome mode). Genome binning from assembled contigs > 2 kbp was performed by running three tools MaxBin (Wu et al., 2016), MetaBAT (Kang et al., 2019), and CONCOCT (Alneberg et al., 2014) using their default settings. Raw genome bins resulting from the three approaches were combined, followed by a selection of the best genome for each genome set using the bin_refinement module in metaWRAP (v1.2) (Uritskiy et al., 2018). During the bin refinement, we applied CheckM_lineage (v1.0.12) (Parks et al., 2015) to evaluate completeness and contamination for each bin. The draft genomes with >50% completeness and <10% contamination were retained for further analysis. Metagenome-assembled genomes (MAGs) were treated with dRep software (Olm et al., 2017) to dereplicate the MAGs by an average nucleotide identity (ANI) threshold of 95% (dereplicate -p 40 -comp 50 -con 10 -pa 0.95 -sa 0.95 -l 10000 -nc 0.30). The taxonomic position of the genomes was identified using genome taxonomy database (GTDB)-tk v0.2.2 (Chaumeil et al., 2020), as well as the calculation of relative evolutionary distance (RED). Coding regions (CDS) for individual MAGs were predicted using Prodigal (version v2.6.3) (Hyatt et al., 2010) with option “-p meta.” Annotation of CDSs was performed using KofamScan (version 1.1.0) (Aramaki et al., 2019) and using Blastp (E value = 1e-5) against the Clusters of Orthologous Gene (COG) and NCBI_nr databases. The results were visualized using the heatmap package of the R platform.

### Phylogenetic Tree Construction

For phylogenomic analysis of Thiohalomonadales genomes, 43 conserved proteins were obtained by the CheckM program with default settings (Katoh et al., 2009) and were used for alignment with Mafft (v7.453, setting: –maxiterate 1000-localpair) (Katoh and Standley, 2013), followed by a further optimization with trimAl (v1.4) (Capella-Gutiérrez et al., 2009). A maximum likelihood (ML) tree was reconstructed using IQ-TREE (v1.6.10) (Nguyen et al., 2015) with the “LG + F + R6” model (Price et al., 2009). Bootstrap values were calculated based on 1,000 replicates.

## Results

### Mineral Composition of the Southwest Indian Ridge Hydrothermal Chimneys

The “Longqi” hydrothermal area is located in the Southwest Indian Ridge. Two sulfide samples, D96S and D100S, were collected from the outer wall of active hydrothermal chimneys on 14 January and 4 February 2015 by the “Jiaolong” submersible (Figure 1 and Supplementary Table 1) in dives nos. 96 and 100, respectively. The chimney rocks were about 10 cm away from the vents. Strong seepage from the hydrothermal vent could be observed. The temperature of the hydrothermal fluid was recorded to be 362 and 365°C for the vents near D96S and D100S, respectively, while the bottom seawater was ∼2.2°C. The pH of the fluid from the two vents was between 3.47 and 3.58. The surface of the D96S sample is gray and has been partially oxidized to grayish brown, while it was light yellow inward with metallic luster (Supplementary Figure 1). There was no megafauna attached to these chimneys. An XRD analysis revealed that D95S and D100S were exclusively composed of sphalerite (ZnS) with a small amount of pyrite (FeS_2_) (<1%) (Supplementary Figure 2). Oxidized minerals were not present in the XRD results, which is in contrast to the dominance of copper-containing chalcopyrite (CuFeS_2_) and kusachiite (CuBi_2_O_4_) in inactive hydrothermal sediments of SWIR (Cao et al., 2014).

### Analysis of Prokaryotic Community Structure

**FIGURE 2:**
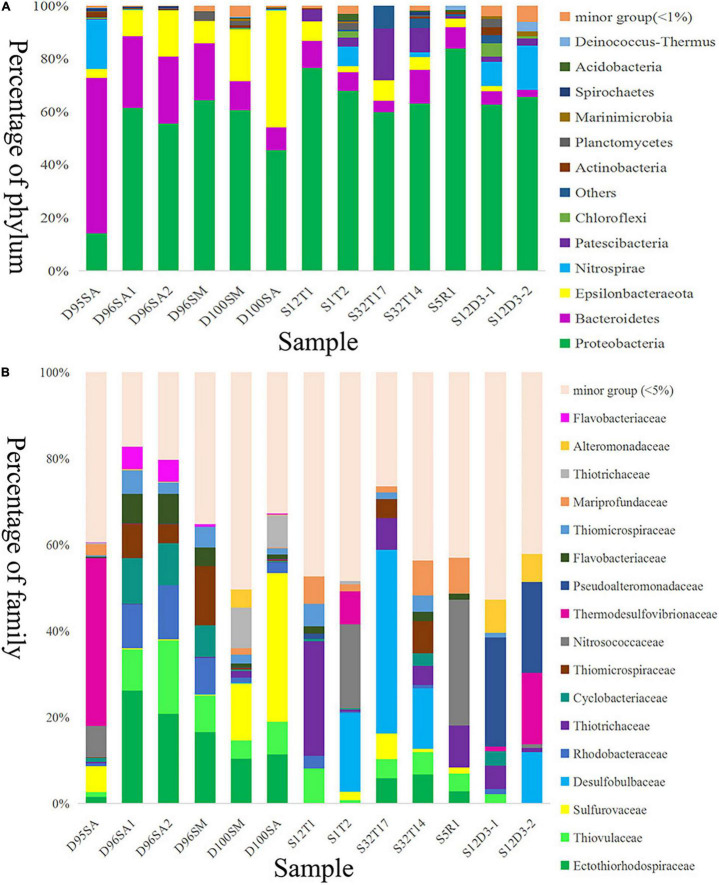
Microbial community structures of hydrothermal samples. Taxonomic classification of operational taxonomic units (OTUs) at 97% similarity was performed by comparing with reference sequences of SILVA database v138. The microbial community structures were shown at phylum **(A)** and family **(B)** levels. D100SA, D96SA1, D96SA2, and D95SA refer to 16S rRNA gene amplicons; D100SM and D96SM are 16S miTags. The other samples are referred to Supplementary Table 2.

**FIGURE 3:**
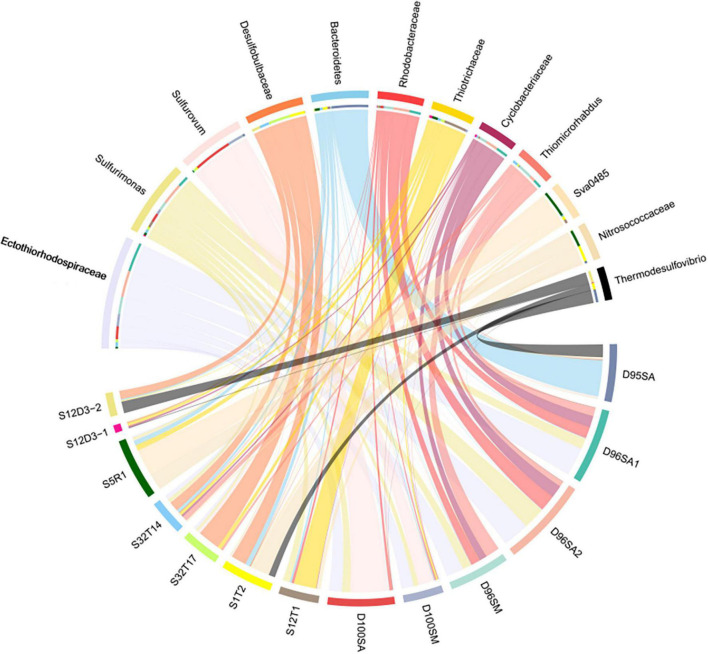
The chord diagram chart showing the distribution of dominant genera. The analysis was performed using the microbial communities at the genus level. D100SA, D96SA1, D96SA2, and D95SA refer to 16S rRNA gene amplicons; D100SM and D96SM are 16S miTags obtained from the metagenomes. The other samples are referred in Supplementary Table 2.

**FIGURE 4:**
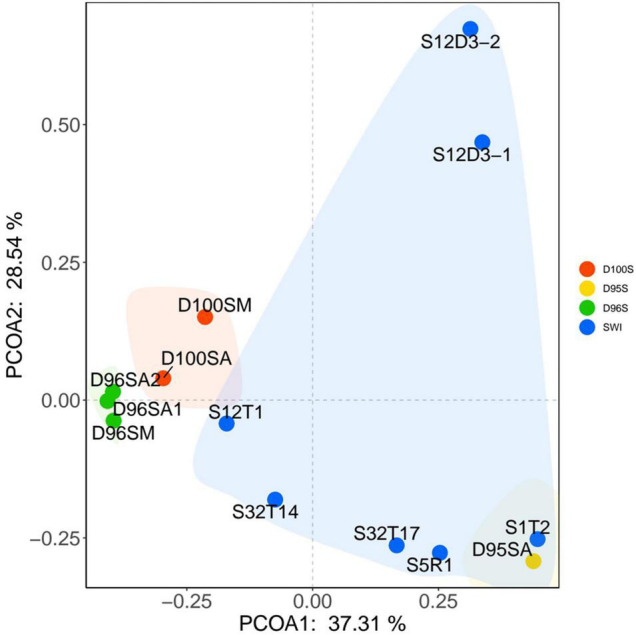
The principal component analysis (PCoA) analysis of microbial communities. The PCoA analysis was performed using the microbial communities at family level. The community structures were based on the classification of 16S miTags (D100SM, D96SM, and the other SWIR reference samples) and 16S rRNA gene amplicons (D96SA1, D96SA2, D100SA, and D95SA) (Supplementary Table 2).

Prokaryotic communities in our chimney samples were examined by sequencing and analysis of 16S rRNA gene amplicons and 16S rRNA gene tags in metagenomic reads (16S miTags), and were then compared with those of the sulfide samples from other SWIR sites (Supplementary Table 1; Dong et al., 2021). There are a total of 30,189 qualified 16S rRNA gene amplicons for the characterization of microbial communities. They were then clustered with 97% similarity into 6,994 OTUs (Supplementary Table 2). Proteobacteria dominated all the SWIR sulfide samples of this study (Figure 2A), except for D95S, in which Bacteroidetes (20%) were the most prevalent phylum. Epsilonbacteraeota was particularly enriched in the chimney samples, and Archaea was not present in these communities. At the family level, *Ectothiorhodospiraceae* (average 11.9%) and *Thiovulaceae* (average 6.8%) were the most abundant families in both the 16S rRNA gene amplicon- and miTag-based communities of the D96S sample. *Ectothiorhodospiraceae* is significantly more abundant in the active chimney samples (D96S and D100S) than in other SWIR hydrothermal sediments using the *U*-test (*p* < 10^–10^). In contrast, D100S contains a high proportion (7.7–19.1%) of *Sulfurovaceae*, which outcompetes *Ectothiorhodospiraceae* (∼6.2%) and *Thiovulaceae* (∼3.2%) as the dominant SOB (Figure 2B). Sulfur reducing bacteria (SRB) represented by *Desulfobulbaceae* and *Thermodesulfovibrionaceae* are abundantly present only in D95S and the reference SWIR sulfide samples. *Thermodesulfovibrionaceae* (Nitrospirae) occupied 6.3% of the community in D95S. The distinct distribution of *Ectothiorhodospiraceae* and *Desulfobulbaceae* between active chimney samples (D96S and D100S) and other hydrothermal samples from the SWIR was further illustrated by a chord diagram (Figure 3). The plotting further revealed special enrichment of Thiotrichaceae as the major SOB in inactive hydrothermal sediments, particularly in the S12T1 sample. A PCoA plot separated the microbial communities of D96S and D100S from the references and D95S. For D96S and D100S, the community structures revealed by the 16S rRNA gene amplicon and miTags were consistent with respect to the PCoA clustering (Figure 4).

### Metagenomics Analysis of Dominant Species Inhabiting Active Hydrothermal Chimney

**FIGURE 5:**
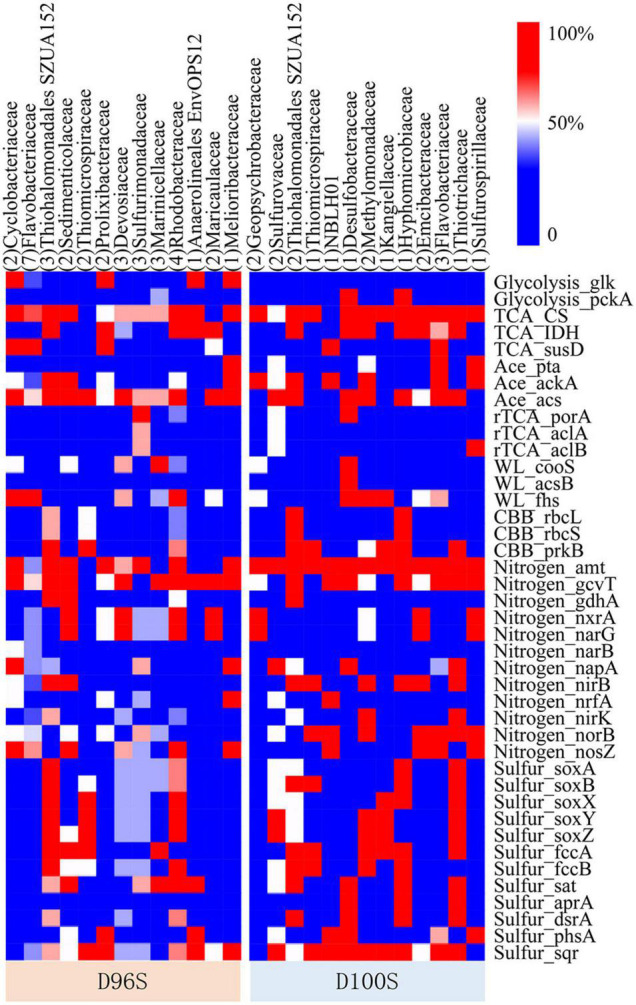
Frequency of functional genes identified in metagenome-assembled genomes (MAGs) belonging to different families. The functional genes of 57 good-quality MAGs were identified by searching against Kyoto Encyclopedia of Genes and Genomes (KEGG) database. The number of MAGs in each genome taxonomy database (GTDB) family was included in the round brackets. The functions of the genes are shown in Supplementary Table 5. The full annotation of KEGG functions is shown in Supplementary Table 6.

To understand the ecological role of microbial inhabitants in the active hydrothermal vents, we obtained 12.1 Gb raw Illumina Miseq paired-end sequencing data (2 × 300 bp) and retained 8.3 Gb clean data for subsequent metagenomics work of D96S and D100S (Supplementary Table 3). After assembly and genome binning, a total of 82 MAGs were retrieved from D96S and D100S metagenomic assemblies (Supplementary Table 4), among which 57 MAGs were of good quality (completeness > 80% and contamination < 5%). Taxonomic classification of the MAGs against GTDB indicates that they were affiliated with 12 prokaryotic phyla mainly composed of Proteobacteria (*n* = 39), Bacteroidota (*n* = 20), Campylobacterota (*n* = 7), and Desulfobacterota (*n* = 5; Supplementary Table 4). The abundant *Ectothiorhodospiraceae* in D96S and D100S identified by 16S rRNA gene amplicon sequencing were not present in the taxa of the MAGs. By searching the 16S rRNA gene amplicons of *Ectothiorhodospiraceae* against those extracted from the MAGs (>97% similarity), we reassigned *Ectothiorhodospiraceae* to the orders Thiohalomonadales SZUA-152 (*n* = 8) and SZUA-140 (*n* = 1) of Gammaproteobacteria in GTDB taxonomy. In a phylogenomics tree, these MAGs were clustered with the genomes obtained from hypersaline soda lake sediment, ground water, and other deep-sea hydrothermal vents, such as Mid-Atlantic Ridge, East Pacific Rise, and Lau Basin of Tonga (Figure 5). Three phylogenetic groups were formed with most of our MAGs in Thiohalomonadales SZUA-152, a new order in GTDB.

**FIGURE 6:**
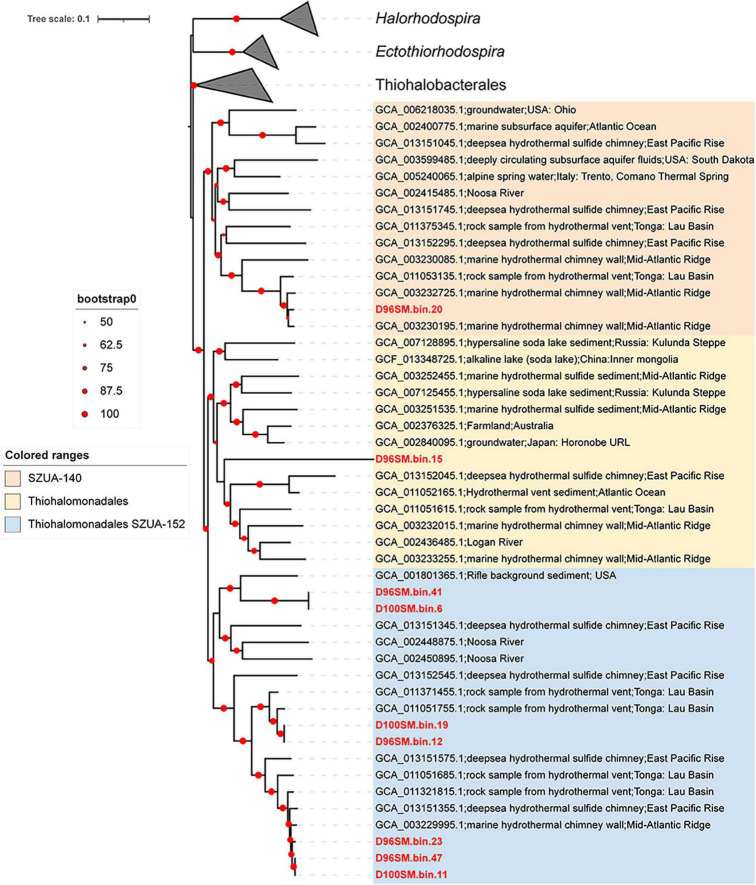
Phylogenomics tree of Thiohalomonadales from the Southwest Indian Ridge (SWIR) hydrothermal chimney samples. The maximum likelihood (ML) phylogenomics tree was constructed using concatenated alignment of 43 conserved proteins extracted from Thiohalomonadales MAGs and reference genomes from GTDB database. The information of the MAGs in red from this study is shown in Supplementary Table 4.

On the basis of Kyoto Encyclopedia of Genes and Genomes (KEGG) gene annotation of the 57 good-quality MAGs, the functional genes for carbon, nitrogen, and sulfur metabolisms mediated by the major families of the chimney inhabitants were examined to predict their ecological functions. Hydrothermal vents discharge large amounts of hydrogen, methane, and hydrogen sulfide to deep waters (Jannasch and Mottl, 1985; Kawagucci et al., 2010; Dick, 2019). Thiosulfate oxidizing genes *soxABXYZ*, and sulfide oxidizing genes *dsrAB*, *fccAB*, and *sqr* were found in ≥50% of the MAGs for Thiohalomonadales (Gammaproteobacteria), *Thiomicrospiraceae* (Gammaproteobacteria), and *Rhodobacteraceae* (Alphaproteobacteria) MAGs from D96S; and in Thiohalomonadales, *Sulfurovaceae* (Campylobacterota), *Hyphomicrobiaceae* (Alphaproteobacteria), and *Thiotrichaceae* (Gammaproteobacteria) MAGs from D100S (Figure 6). In the periplasmic space, FccAB catalyzes oxidation of sulfide to elemental sulfur with electrons being transferred to cytochrome c; the SQR enzyme oxidizes sulfide to polysulfide (Schutz et al., 1999). The DsrAB encoded by the SOB carry out sulfide oxidation to sulfite (Anantharaman et al., 2014; Dong et al., 2021). This indicates the capacity of sulfide oxidation to S^0^, polysulfide or sulfate by these SOB bacteria inhabiting our active chimney samples under different fluxes of electron acceptors. The *Rhodobacteraceae* MAGs also harbor nitrate reduction genes *narG*, suggesting the coupling of nitrate reduction and sulfide oxidation. *Sulfurimonadaceae* and *Sulfurovaceae* encoded the reverse tricarboxylic acid (rTCA) pathway for autotrophic CO_2_ fixation. The other SOB, with the exception of *Thiotrichaceae*, may rely on the Calvin–Benson–Bassham (CBB) pathway for autotrophs, as ribulose-1,5-bisphosphate carboxylase (RuBisCO) genes *rbcLS* were found in their MAGs. Wood-Ljungdahl pathway as an alternative autotrophic process was only encoded by *Desulfobacteraceae* MAGs. Acetate metabolism is highly required by the bacterial inhabitant, as evidenced by the prevalence of acetate assimilatory genes *pta*, *acs*, and *ackA* in these MAGs. Genomics data also indicate that Gammaproteobacteria might use hydrogen released from the vents as alternative energy sources for carbon fixation and metabolic activities as indicated by the detection of [NiFe]-hydrogenase genes.

### Metabolism Potentials of Thiohalomonadales

**FIGURE 7:**
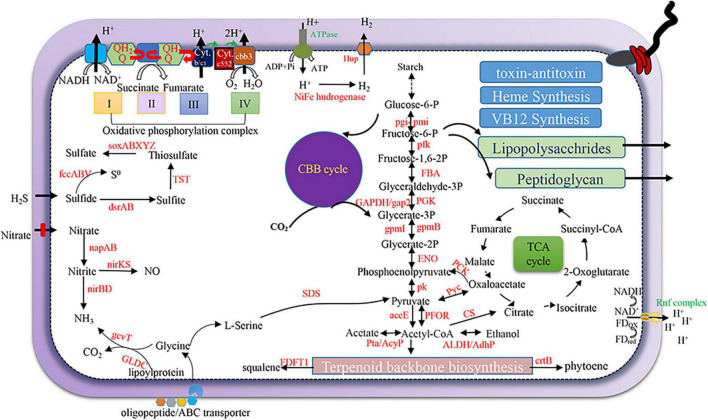
Schematic metabolism of Thiohalomonadales (sulfur oxidizing bacteria) SOB. The metabolic pathways were predicted and depicted based on gene annotation of high-quality MAGs of Thiohalomonadales against KEGG and National Center for Biotechnology Information (NCBI)_nr databases.

We next examined the metabolic potentials and adaptive strategy of the dominant SOB, Thiohalomonadales, from our active hydrothermal vents using the annotation result of their five high-quality MAGs. The Thiohalomonadales MAGs harbor a complete set of sulfur-oxidizing genes, such as *soxABXYZ*, *fccABV, sqr*, *rdsrAB*, and *TST*, that may yield S^0^, polysulfide, or sulfate (Figure 7). Nitrate assimilatory reduction may occur in the Thiohalomonadales as the MAGs contain *napAB* periplasmic nitrate reduction genes and nitrite reduction genes (*nirBD*) that are involved in ammonia production. Thiohalomonadales might also rely on the CBB pathway for CO_2_ fixation. Thiohalomonadales MAGs contain *cbb3* genes coding for phosphorylation respiration complex IV to overcome low oxygen conditions. Considering the high hydrogen content of the hydrothermal fluids, the microbes on the chimney are expected to be able to utilize hydrogen as an energy source. [NiFe] hydrogenase genes were present in the MAGs, along with *hup* genes responsible for the uptake and/or export of hydrogen. The Rnf complex as a cross-membrane proton pumping machine for the balance of cytoplasmic pH was encoded by the MAGs (Buckel and Thauer, 2013).

We identified all the related genes encoding the enzymes that participate in terpenoid backbone biosynthesis. Using geranylgeranyl diphosphate, Thiohalomonadales might generate phytoene, since the *crtB* gene encoding 15-cis-phytoene synthase was identified in the MAGs. The CrtB protein of Thiohalomonadales is most similar (82%) to a homolog of Thiotrichaceae from a subseafloor aquifer. Phytoene is a precursor of zeta-carotene and can be catalyzed by phytoene desaturase CtrI for carotene production (Lang et al., 1994). However, *ctrI* gene is missing from the MAGs, and the capacity of carotene biosynthesis by these SOB is thus questioned. The *crtB* gene was also present in the MAGs from RBG-16-57-12, Mariprofundaceae, Thiohalomonadales SZUA-140, and Melioribacteraceae. Most of these *crtB-*bearing MAGs also contain a farnesyl-diphosphate farnesyltransferase coding gene (*FDFT1*) that functions in squalene synthesis using farnesyl diphosphate.

In the Thiohalomonadales MAGs, a cph2 type bacteriophytochrome coding gene was identified to be 45% similar to a homolog from *Pseudomonas aeruginosa*. However, the putative bacteriophytochrome is featured with GGDEF and EAL domains but lacks GAF and PHY domains, indicating a discovery of a novel or malfunctional bacteriophytochrome (Gourinchas et al., 2019). The co-factor of bacteriophytochrome is biliverdin with a tetrapyrrolic structure. Heme synthesis pathway and biliverdin-producing heme oxygenase have been identified in the MAGs, which are vital for the potential function of bacteriophytochrome under red and far-red lights for Thiohalomonadales species (Vuillet et al., 2007; Gourinchas et al., 2019).

## Discussion

### Discovery of Novel Microbiomes in Active Hydrothermal Chimneys

In this study, the “Jiaolong” manned submersible with robotic precision was employed to locate the vent structure of sulfide chimneys in the active SWIR hydrothermal areas. Using these chimney samples, we report the genomes of dominant microbial species and the prevalence of distinct SOB affiliated with Campylobacterota, Gammaproteobacteria, and Alphaproteobacteria inhabiting the sulfide chimney, all of which have been reported in deep-sea cold seeps recently (Li et al., 2021). The microbial community in the active SWIR hydrothermal vents was remarkably different from those from the SWIR inactive hydrothermal sulfide sediments (Cao et al., 2014; Dong et al., 2021) and others DSHVs (Dick, 2019; Hou et al., 2020; Zeng et al., 2021). Culturable Thiohalomonadales SOB, such as *Thiohalomonas denitrificans* had been isolated from the hydrothermal vents of the Suiyo Seamount in the Pacific Ocean (Mori et al., 2015). In addition, this study demonstrates their distribution in the DSHVs located in the Mid-Atlantic Ridge, East Pacific Rise, and Lau Basin. Given the finding of their relatives in other worldwide sites, the dominance of this order in DSHVs was, however, not reported previously (Flores et al., 2011; Urich et al., 2014; Meier et al., 2017). Dominant SOB differed even between D96S and D100S, owing to different combinations of Thiohalomonadales, *Thiomicrorhabdus*, *Sulfurovum*, and *Sulfurimonas*. This suggests a high diversity of SOB among active hydrothermal vents. Our genomics data predict other potential SOB, such as Hyphomicrobiaceae, Thiotrichaceae (represented by Beggiatoa), and Rhodobacteraceae in our samples, which have been rarely reported in other DSHVs. Considering the highly variable microenvironments adjacent to DSHVs, the composition of SOB inhabitants is predicted to be slightly diversified as reported for D96S and D100S by this study. In the late stages of hydrothermal vents, weak seepage of reducing fluids allows soaking of sulfide with oxic bottom water, which will gradually result in sulfide mineral oxidation and subsequent microbially mediated reduction processes (Li et al., 2017). The microbiomes of D96S and D100S were solely constituted by SOB, which is an indicator of the initial stage of a hydrothermal ecosystem. This has not been noticed in the SWIR and even global DSHVs.

The strong hydrothermal venting probably creates a reducing environment that covers the chimney, prohibiting oxidation of the chimney sulfide in this study. The mineral components in our samples indicate an early stage of the chimney formation at D100S, as there were not any oxidized mineral components. As a result, we could not detect SRB in D100S, while in contrast, prevalent *Thermodesulfovibrionaceaea* (SRB) was present in D95S. Although the sulfate concentration and nutrients of the samples were not analyzed due to their contact with sea water, we speculate that sulfate from sea water and produced by SOB might probably fuel the SRB in D95S. It seems that *Sulfurovaceae*, Thiohalomonadales, and *Thiovulaceae* were distributed differently in the hydrothermal chimneys possibly due to selection of different SOB families by local environmental variants. For example, *Sulfurovum* is more tolerant to oxygen (Meier et al., 2017) and might prefer a more oxic chimney with lower impact of hydrothermal fluid. In this study, Thiohalomonadales probably employed *cbb3* to cope with low oxygen; however, hypoxic exposure may also impair anaerobic and microaerobic microbial inhabitants (Lu and Imlay, 2021). We found that squalene as a bacterial hopanoid was likely synthesized by Thiohalomonadales, which is probably an efficient mechanism to scavenge single oxygen that may damage cell lipid by peroxidation (Kohno et al., 1995). The microbes in D96S and D100S have probably evolved to contain distinct gene profiles for adaptation to environmental changes due to the dynamics of gas and metal fluxes near active hydrothermal vents.

### Photolithoautotrophic Potential of Sulfur-Oxidizing Thiohalomonadales

*Ectothiorhodospiraceae* was previously a family of Chromatiales, but was recently reclassified into a new family of Thiohalomonadales. Members of the *Ectothiorhodospiraceae* are versatile in metabolisms, including photolithotrophic, photoheterotrophic, chemoheterotrophic, chemolithotrophic, and methylotrophic bacteria using various electron acceptors, such as nitrite, sulfur compounds, and arsenite (Hallberg et al., 2011; Slobodkina et al., 2016). So far, all isolates from hydrothermal fields belonging to this family are sulfur-oxidizing bacteria (Imhoff, 2006; Mori et al., 2015; Slobodkina et al., 2016). In this study, we discovered three phylogenetic groups in Thiohalomonadales belonging to SZUA152, SZUA140, and an unclassified clade. *Ectothiorhodospiraceae* members are able to capture red/far-red light for anaerobic photosynthesis, by which sulfide is oxidized to elemental sulfur and deposited in periplasm for further oxidation to sulfate (Imhoff, 2006). Bacteriophytochromes are photosensitive proteins employed by bacteria to capture red/far-red light to mediate growth (Vuillet et al., 2007; Gourinchas et al., 2019). At hydrothermal vents with a temperature of ∼350°C, most of the ambient light was detected to fall into near-infrared spectra between 750 and 1,050 nm (Dover et al., 1994). A recent report showed the promoted growth of a bacterial cultivation isolated from the Western Pacific DSHV under stimulation of infrared light at 940 nm (Liu et al., 2021). This is highly supported by a recent report that showed the presence of proteorhodopsin synthesis genes and photoautotrophic microbial groups in some metagenomes of active hydrothermal vents of SWIR (Chen et al., 2022). If the bacteriophytochromes and cofactor biliverdin are functional in Thiohalomonadales, they can probably sense red/near-infrared light from the hydrothermal vents to activate photosynthesis of Thiohalomonadales. In photosynthetic and non-photosynthetic organisms, carotenoids are synthesized to prevent the photooxidative damage (Glaeser and Klug, 2005). In the present study, although the gene coding for the enzyme catabolizing the final step of zeta-carotenoid was not found, there are still perhaps unknown genes responsible for carotenoid synthesis in the genomes. Aside from serving as light sensors, some bacteriophytochromes can be redox sensors to monitor the environmental changes, particularly in the environment approximate to hydrothermal vents where hot reductive fluid meets cold oxic sea water. Whether Thiohalomonadales can synthesize the *bona fide* bacteriophytochromes for photoautotrophy warrants future experimental efforts using cultivated strains of this order from DSHVs.

Overall, we identified an unusual, uncultivated sulfur-oxidizing bacterial group that is particularly prevalent in active hydrothermal chimneys. Their potential capacity of sulfur oxidization and photoautotrophic lifestyle casts lights on the possible relationship with ancient lineages inhabiting the early earth with similar hydrothermal environments billions of years ago (Lunine, 2006). The finding of this SOB group can probably provide insights into the future evolutionary study of ancient microbial lineages under the climate change in the long history of the Earth.

## Data Availability Statement

The datasets presented in this study can be found in online repositories. The names of the repository/repositories and accession number(s) can be found in the article/Supplementary Material.

## Author Contributions

H-YB and YW conceived the study and wrote the manuscript. H-GC, P-FZ, and Y-LZ performed the experiments. YW, H-YB, P-FZ, and Y-LZ analyzed the data and summarized the results. J-TL critically revised the manuscript. All authors contributed to the article and approved the submitted version.

## Conflict of Interest

The authors declare that the research was conducted in the absence of any commercial or financial relationships that could be construed as a potential conflict of interest.

## Publisher’s Note

All claims expressed in this article are solely those of the authors and do not necessarily represent those of their affiliated organizations, or those of the publisher, the editors and the reviewers. Any product that may be evaluated in this article, or claim that may be made by its manufacturer, is not guaranteed or endorsed by the publisher.

## Footnotes

1
http://www.bioinformatics.babraham.ac.uk/projects/fastqc/
