# Supplementary material for: Metagenomics Reveals Dominant Unusual Sulfur Oxidizers Inhabiting Active Hydrothermal Chimneys From the Southwest Indian Ridge
Source: Front Microbiol. 2022 May 25;13:861795. doi: 10.3389/fmicb.2022.861795 (PMC9174799; doi:10.3389/fmicb.2022.861795)

**Table S1. Depth and location of SWIR sulfide samples.**

| Sample | Depth(m) | Latitude | Longitude | Vent temp. | pH |
| --- | --- | --- | --- | --- | --- |
| D95S | 2789 | 37.77°S | 49.63°E | - | - |
| D96S | 2768 | 37.78°S | 49.65°E | 362 | 3.47 |
| D100S | 2755 | 37.78°S | 49.65°E | 365 | 3.58 |
| S5R1 | 2742 | 37.78°S | 49.65°E | - | - |
| S12D3-1 | 2328 | 37.78°S | 49.65°E | - | - |
| S12D3-2 | 2328 | 37.78°S | 49.65°E | - | - |
| S12T1 | 2820 | 37.78°S | 49.65°E | - | - |
| S1T2 | 2878 | 37.78°S | 49.65°E | - | - |
| S32T14 | 1744 | 38.10°S | 50.76°E | - | - |
| S35T17 | 2738 | 38.30°S | 51.08°E | - | - |

D95S, D96S, and D100S were obtained from chimneys for this study. The other samples were sulfide deposits of SWIR (Dong et al., 2021).

**Table S2. Analysis of 16S rRNA gene amplicons and miTags.**

| Sample | Reads-source | Barcode | No. qualified amplicons | No. OTUs |
| --- | --- | --- | --- | --- |
| S5R1 | miTag | - | 254 | 237 |
| S32T17 | miTag | - | 265 | 210 |
| S12D3-1 | miTag | - | 335 | 296 |
| S12D3-2 | miTag | - | 314 | 245 |
| S32T14 | miTag | - | 397 | 379 |
| S12T1 | miTag | - | 414 | 372 |
| S1T2 | miTag | - | 628 | 522 |
| D100SM | miTag | - | 5305 | 2608 |
| D96SM | miTag | - | 7871 | 3340 |
| D100SA | Amplicon | GCCTAA | 3451 | 1204 |
| D96SA1 | Amplicon | TGGTCA | 6078 | 1850 |
| D96SA2 | Amplicon | CACTGT | 8281 | 1905 |
| D95SA | Amplicon | ACATCG | 12379 | 2035 |

**Table S3. Sequencing and assembly result of metagenomes from the Longqi hydrothermal area of the Southwest Indian Ocean.**

| Sample |  | Raw data [Gbp] | No. clean reads | Clean data [Gbp] | Assembly  data  [Gbp] | Assembled contigs(>2kb) [Mbp] |  |
| --- | --- | --- | --- | --- | --- | --- | --- |
| D96SM |  | 8.11 | 21613618 | 5.64 | 0.35 | 150 |  |
| D100SM |  | 4.09 | 9858654 | 2.61 | 0.54 | 259 |  |

**Table S4. Summary of MAGs**

| D96S MAG | %Completeness | %Conta  mination | Genome size (Mbp) | N50 (kb) | No. of contigs | No. of genes | Coverage | RED value | Taxonomy |
| --- | --- | --- | --- | --- | --- | --- | --- | --- | --- |
| bin.1 | 84.91 | 1.6 | 3.04 | 33.36 | 116 | 2770 | 2.82 | 0.79 | Bac;Bac;Bac; Cytophagales; Cyclobacteriaceae; |
| bin.10 | 99.46 | 0 | 3.24 | 76.27 | 66 | 2743 | 3.24 | 0.83 | Bac;Bac;Bac; Bacteroidales; NBLH01; _; |
| bin.11 | 91.69 | 0.59 | 2.25 | 75.55 | 42 | 2024 | 3.81 | 0.89 | Bac;Bac;Bac; Flavobacteriales; Flavobacteriaceae; _CG1-02-35-72; |
| bin.12 | 83.75 | 1.43 | 3.12 | 35.65 | 122 | 2930 | 31.74 | 0.83 | Bac;Pro;Gammapro; Thiohalomonadales_A; SZUA-152; _; |
| bin.13 | 98.19 | 1.78 | 2.95 | 47.48 | 98 | 2807 | 8.78 | 0.78 | Bac;Pro;Gammapro; Chromatiales; Sedimenticolaceae; _; |
| bin.14 | 57.73 | 2.45 | 2.44 | 16.67 | 160 | 2220 | 14.86 | 0.80 | Bac;Bac;Bac; Cytophagales; Cyclobacteriaceae; _; |
| bin.15 | 80.48 | 1.21 | 1.92 | 21.56 | 96 | 1894 | 18.60 | 0.96 | Bac;Pro;Gammapro; Thiomicrospirales; Thiomicrospiraceae;  _Thiomicrorhabdus; |
| bin.16 | 62.85 | 1.72 | 3.10 | 4.77 | 719 | 3166 | 1.48 | 0.92 | Bac;Bac;Bac; Bacteroidales; Prolixibacteraceae; _Draconibacterium; |
| bin.18 | 80.64 | 3.89 | 1.83 | 16.27 | 117 | 1849 | 3.99 | 0.90 | Bac;Pro;c__Alphaproteobacteria; Rhizobiales; Devosiaceae; _; |
| bin.19 | 63.11 | 6.73 | 2.18 | 10.68 | 210 | 1967 | 1.72 | 0.95 | Bac;Bac;Bac; Bacteroidales; F082; _B6-G9; |
| bin.2 | 50.43 | 0 | 2.01 | 3.62 | 569 | 2380 | 1.32 | 0.81 | Bac;p__Spirochaetota;c__Spirochaetia; Spirochaetales; B11-G9; _; |
| bin.20 | 96.45 | 0.35 | 3.36 | 61.19 | 76 | 3066 | 11.76 | 0.98 | Bac;Pro;Gammapro; SZUA-140; SZUA-140; _SZUA-76; |
| bin.21 | 54.2 | 2.20 | 1.55 | 4.09 | 404 | 1875 | 1.38 | 0.95 | Bac;Pro;c__Alphaproteobacteria; Caulobacterales;  Hyphomonadaceae; _Henriciella; |
| bin.22 | 96.58 | 4.48 | 2.05 | 35.06 | 100 | 2108 | 8.39 | 0.95 | Bac;Cam;Campylo; Campylobacterales; Sulfurimonadaceae;  _Sulfurimonas; |
| bin.23 | 83.93 | 2.64 | 3.13 | 37.84 | 102 | 2948 | 35.65 | 0.97 | Bac;Pro;Gammapro; Thiohalomonadales_A; SZUA-152;  _SZUA-152; |
| bin.24 | 51.17 | 0 | 3.01 | 13.90 | 218 | 2605 | 2.23 | 0.93 | Bac;Bac;Bac; Bacteroidales; Prolixibacteraceae;  _Draconibacterium; |
| bin.25 | 91.77 | 2.20 | 3.07 | 19.44 | 213 | 2899 | 3.48 | 0.91 | Bac;Bac;Bac; Flavobacteriales; Flavobacteriaceae; _; |
| bin.26 | 88.26 | 3.54 | 2.60 | 13.77 | 235 | 2647 | 2.00 | 0.91 | Bac;Pro;c__Alphaproteobacteria; Rhizobiales; Rhizobiaceae;  _GCA-2401155; |
| bin.27 | 97.23 | 1.67 | 2.68 | 60.77 | 67 | 2584 | 3.11 | 0.89 | Bac;Pro;c__Alphaproteobacteria; Rhizobiales; Devosiaceae; _; |
| bin.28 | 87.19 | 1.22 | 1.68 | 25.83 | 71 | 1776 | 3.97 | 0.94 | Bac;Cam;Campylo; Campylobacterales; Sulfurimonadaceae;  _Sulfurimonas; |
| bin.29 | 97.95 | 1.93 | 4.62 | 40.19 | 141 | 4192 | 3.00 | 0.79 | Bac;Ver;c__Verrucomicrobiae; Verrucomicrobiales; DEV007; _; |
| bin.3 | 73.9 | 7.65 | 2.62 | 6.60 | 472 | 2687 | 1.78 | 0.85 | Bac;Pro;Gammapro; Xanthomonadales; Marinicellaceae; _; |
| bin.30 | 95.89 | 1.21 | 2.99 | 45.65 | 91 | 3114 | 3.12 | N/A | Bac;Pro;c__Alphaproteobacteria; Rhodobacterales;  Rhodobacteraceae; _Marinosulfonomonas;  Marinosulfonomonas sp002732995 |
| bin.31 | 67.9 | 4.96 | 2.00 | 5.09 | 430 | 2303 | 1.55 | 0.90 | Bac;Pro;c__Alphaproteobacteria; Rhizobiales; Devosiaceae; _; |
| bin.32 | 83.87 | 1.60 | 1.97 | 51.69 | 55 | 2012 | 2.85 | 0.95 | Bac;Cam;Campylo; Campylobacterales; Sulfurimonadaceae;  _Sulfurimonas; |
| bin.33 | 88.33 | 0.95 | 2.83 | 40.28 | 112 | 2635 | 9.45 | 0.99 | Bac;Bac;Bac; Flavobacteriales; Flavobacteriaceae;  _GCA-002733185; |
| bin.34 | 84.65 | 2.79 | 2.55 | 22.24 | 130 | 2640 | 6.56 | 0.97 | Bac;Pro;c__Alphaproteobacteria; Rhodobacterales;  Rhodobacteraceae; _UBA3077; |
| bin.35 | 66.39 | 3.305 | 2.05 | 5.28 | 436 | 2256 | 1.48 | 0.91 | Bac;Pro;Gammapro; Xanthomonadales; Marinicellaceae; _; |
| bin.36 | 60.7 | 1.75 | 2.04 | 12.32 | 215 | 1999 | 1.92 | 0.90 | Bac;Bac;Bac; Flavobacteriales; Flavobacteriaceae; _QNYL01; |
| bin.37 | 56.37 | 0 | 1.22 | 8.62 | 171 | 1300 | 2.70 | 0.96 | Bac;Pro;Gammapro; Thiomicrospirales; Thiomicrospiraceae;  _Thiomicrorhabdus; |
| bin.38 | 93.6 | 0.88 | 2.66 | 146.39 | 31 | 2507 | 6.12 | 0.74 | Bac;Pro;Gammapro; Ga0077554; _; _; |
| bin.39 | 55.77 | 7.60 | 2.58 | 3.47 | 756 | 2728 | 1.32 | 0.66 | Bac;Mar;c__UBA8477; UBA8477; _; _; |
| bin.4 | 93.72 | 6.28 | 3.23 | 45.45 | 111 | 3070 | 6.64 | 0.98 | Bac;Pro;Gammapro; Xanthomonadales; SZUA-36; _SZUA-51; |
| bin.41 | 98.59 | 1.93 | 2.53 | 47.68 | 73 | 2381 | 2.93 | 0.79 | Bac;Pro;Gammapro; Thiohalomonadales_A; RBG-16-57-12; _; |
| bin.42 | 82.87 | 0.81 | 2.56 | 23.51 | 168 | 2498 | 3.22 | 0.99 | Bac;Pro;Gammapro; Xanthomonadales; SZUA-36; _SZUA-51; |
| bin.43 | 53.55 | 1.07 | 2.32 | 5.89 | 450 | 2262 | 1.59 | 0.82 | Bac;Plan;c__UBA8742; UBA2392; UBA2392; _; |
| bin.44 | 94.74 | 2.20 | 3.46 | 68.79 | 67 | 3373 | 5.06 | 0.98 | Bac;Pro;c__Alphaproteobacteria; Rhodobacterales;  Rhodobacteraceae; _NORP181; |
| bin.45 | 70.18 | 5.07 | 2.03 | 18.87 | 118 | 1866 | 2.29 | 0.85 | Bac;Pro;Gammapro; Xanthomonadales; Marinicellaceae; _; |
| bin.46 | 87.97 | 1.51 | 3.01 | 56.31 | 80 | 2695 | 5.58 | 0.90 | Bac;Bac;Bac; Flavobacteriales; Flavobacteriaceae; _QNYL01; |
| bin.47 | 87.22 | 2.58 | 4.01 | 75.43 | 81 | 3620 | 19.57 | 0.97 | Bac;Pro;Gammapro; Thiohalomonadales_A; SZUA-152;  _SZUA-152; |
| bin.48 | 82.02 | 1.12 | 0.98 | 984.18 | 1 | 927 | 5.30 | 0.82 | Bac;Pate;c__ABY1; Magasanikbacterales; UBA922; _; |
| bin.49 | 94.71 | 2.03 | 3.43 | 28.97 | 163 | 3120 | 2.86 | 0.79 | Bac;Pro;Gammapro; Chromatiales; Sedimenticolaceae; _; |
| bin.5 | 66.42 | 1.46 | 4.62 | 4.18 | 1153 | 4422 | 1.43 | 0.70 | Bac;Hyd;c__Hydrogenedentia; Hydrogenedentiales; ; _; |
| bin.50 | 88.52 | 1.65 | 2.59 | 52.64 | 67 | 2371 | 3.85 | 0.97 | Bac;Bac;Bac; Flavobacteriales; Flavobacteriaceae; _PRS1; |
| bin.51 | 98.21 | 0.59 | 2.73 | 28.93 | 118 | 2445 | 3.43 | 0.81 | Bac;Des;c__Desulfobulbia; Desulfobulbales; BM004; _; |
| bin.52 | 55.15 | 1.91 | 1.46 | 4.17 | 361 | 1663 | 1.37 | 0.85 | Bac;Chl;c__Anaerolineae; Anaerolineales; EnvOPS12; _; |
| bin.53 | 71.27 | 1.95 | 2.03 | 19.21 | 115 | 1930 | 2.35 | 0.98 | Bac;Bac;Bac; Flavobacteriales; Flavobacteriaceae; _LPB0138; |
| bin.6 | 86.39 | 2.72 | 2.27 | 9.92 | 286 | 2376 | 1.83 | 0.94 | Bac;Pro;c__Alphaproteobacteria; Caulobacterales;  Maricaulaceae; _Robiginitomaculum_B; |
| bin.7 | 58.68 | 4.79 | 1.85 | 3.67 | 519 | 2236 | 1.50 | 0.98 | Bac;Pro;c__Alphaproteobacteria; Caulobacterales;  Maricaulaceae; _Robiginitomaculum_A; |
| bin.8 | 96.18 | 2.13 | 2.60 | 80.59 | 51 | 2639 | 12.65 | 0.97 | Bac;Pro;c__Alphaproteobacteria; Rhodobacterales;  Rhodobacteraceae; _UBA3077; |
| bin.9 | 97.2 | 2.23 | 4.25 | 62.74 | 99 | 3552 | 3.46 | 0.94 | Bac;Bac;c__Ignavibacteria; Ignavibacteriales;  Melioribacteraceae; _1-14-2-50-31-20; |
| D100S  MAG | completeness | Contamination | Genome size (Mbp) | N50 (kb) | No. of contigs | No. of genes |  | RED value | Taxonomy |
| bin.1 | 99.35 | 1.29 | 3.43 | 41.48 | 146 | 3253 | Coverage | 0.95 | Bac;Des;c__Desulfuromonadia; Desulfuromonadales;  Geopsychrobacteraceae; _Desulfuromusa; |
| bin.10 | 93.03 | 4.30 | 2.15 | 22.85 | 166 | 2373 | 126.31 | 0.98 | Bac;Cam;Campylo; Campylobacterales; Sulfurovaceae;  _Sulfurovum; |
| bin.11 | 96.24 | 3.46 | 4.44 | 76.10 | 82 | 4084 | 641.44 | 0.97 | Bac;Pro;Gammapro; Thiohalomonadales_A; SZUA-152; _SZUA-152; |
| bin.12 | 70.95 | 0.15 | 1.47 | 4.67 | 346 | 1653 | 150.61 | 0.96 | Bac;Pro;Gammapro; Thiomicrospirales; Thiomicrospiraceae;  _Thiomicrorhabdus; |
| bin.13 | 90.72 | 3.49 | 4.24 | 10.13 | 515 | 3873 | 67.24 | 0.90 | Bac;Bac;Bac; Bacteroidales; NBLH01; _NBLH01; |
| bin.14 | 94.82 | 7.47 | 2.30 | 12.64 | 270 | 2642 | 78.83 | 0.96 | Bac;Cam;Campylo; Campylobacterales; Sulfurovaceae; _Sulfurovum; |
| bin.15 | 92.71 | 1.45 | 4.27 | 19.22 | 332 | 4067 | 136.60 | 0.93 | Bac;Des;c__Desulfobacteria; Desulfobacterales; Desulfobacteraceae;  _Desulfobacula; |
| bin.16 | 96.89 | 2.21 | 4.02 | 50.66 | 116 | 3663 | 86.69 | 0.93 | Bac;Pro;Gammapro; Methylococcales; Methylomonadaceae; _QPIN01; |
| bin.17 | 53.55 | 3.33 | 2.19 | 3.90 | 605 | 2553 | 287.71 | 0.94 | Bac;Pro;Gammapro; Enterobacterales; Kangiellaceae; _GCA-002733465; |
| bin.18 | 50.85 | 0.54 | 1.08 | 3.34 | 325 | 1285 | 58.77 | 0.54 | Bac;p__WOR-3;c__Hydrothermia; ; ; _; |
| bin.19 | 98.76 | 1.70 | 3.74 | 47.52 | 117 | 3471 | 75.70 | 0.83 | Bac;Pro;Gammapro; Thiohalomonadales_A; SZUA-152; _; |
| bin.2 | 96.74 | 1.57 | 2.97 | 14.19 | 302 | 2984 | 1124.72 | 0.77 | Bac;Pro;c__Alphaproteobacteria; Rhizobiales; Hyphomicrobiaceae; _; |
| bin.20 | 83.14 | 2.42 | 2.65 | 7.64 | 416 | 2840 | 94.00 | 0.98 | Bac;Pro;c__Alphaproteobacteria; Sphingomonadales; Emcibacteraceae;  _Emcibacter_A; |
| bin.21 | 99.5 | 0.50 | 2.85 | 197.06 | 40 | 2660 | 84.15 | 0.99 | Bac;Pro;c__Alphaproteobacteria; Sphingomonadales; Emcibacteraceae;  _Emcibacter_A; |
| bin.22 | 95.78 | 1.17 | 2.99 | 46.12 | 103 | 2689 | 164.34 | 0.96 | Bac;Bac;Bac; Flavobacteriales; Flavobacteriaceae; _Lutibacter; |
| bin.23 | 72.37 | 1.72 | 2.52 | 5.05 | 546 | 2794 | 447.96 | 0.94 | Bac;Des;c__Desulfuromonadia; Desulfuromonadales;  Geopsychrobacteraceae; _Geopsychrobacter; |
| bin.24 | 76.35 | 2.79 | 2.90 | 5.40 | 609 | 3012 | 65.66 | 0.81 | Bac;Bac;Bac; Bacteroidales; 4572-128; _; |
| bin.25 | 90.68 | 1.31 | 2.43 | 68.79 | 51 | 2194 | 64.87 | 0.93 | Bac;Bac;Bac; Flavobacteriales; Flavobacteriaceae; _CG1-02-35-72; |
| bin.26 | 93.88 | 1.62 | 2.73 | 27.06 | 143 | 2698 | 140.29 | 0.98 | Bac;Pro;c__Alphaproteobacteria; Caulobacterales; Maricaulaceae;  _Robiginitomaculum_A; |
| bin.27 | 92.61 | 2.12 | 2.85 | 85.59 | 48 | 2727 | 103.86 | 0.93 | Bac;Pro;Gammapro; Thiotrichales; Thiotrichaceae; _Cocleimonas; |
| bin.28 | 77.18 | 1.69 | 1.39 | 5.66 | 280 | 1623 | 644.44 | 0.94 | Bac;Cam;Campylo; Campylobacterales; Hydrogenimonadaceae;  _Hydrogenimonas; |
| bin.29 | 94.59 | 1.35 | 4.86 | 34.01 | 217 | 3776 | 65.13 | 0.76 | Bac;Ver;c__Lentisphaeria; Victivallales; Victivallaceae; _; |
| bin.3 | 82.8 | 3.33 | 3.20 | 9.38 | 438 | 3177 | 160.60 | 0.80 | Bac;Des;c__Desulfobulbia; Desulfobulbales; Desulfobulbaceae; _; |
| bin.4 | 90.77 | 1.44 | 2.74 | 43.56 | 99 | 2488 | 90.04 | 0.98 | Bac;Bac;Bac; Flavobacteriales; Flavobacteriaceae; _Lutibacter; |
| bin.5 | 59.91 | 4.47 | 1.10 | 3.01 | 362 | 1439 | 148.30 | 0.98 | Bac;Pro;c__Zetaproteobacteria; Mariprofundales; Mariprofundaceae;  _Ghiorsea; |
| bin.6 | 99.64 | 1.05 | 2.58 | 59.88 | 64 | 2419 | 80.11 | 0.80 | Bac;Pro;Gammapro; Thiohalomonadales_A; RBG-16-57-12; _; |
| bin.7 | 97.31 | 0.53 | 3.53 | 123.92 | 46 | 2896 | 157.46 | 0.95 | Bac;Bac;Bac; Bacteroidales; F082; _B6-G9; |
| bin.8 | 70.61 | 5.12 | 1.69 | 4.89 | 392 | 2095 | 178.78 | 0.84 | Bac;Cam;Campylo; Campylobacterales; Sulfurospirillaceae; _; |
| bin.9 | 93.96 | 1.92 | 3.50 | 24.13 | 216 | 3388 | 63.07 | 0.93 | Bac;Pro;Gammapro; Methylococcales; Methylomonadaceae; _QPIN01; |

**Table S5. Function of genes in Figure 5.**

| Gene ID | Function |
| --- | --- |
| Glycolysis_glk | glk; glucokinase [EC:2.7.1.2] |
| Glycolysis_pckA | E4.1.1.32, pckA, PCK; phosphoenolpyruvate carboxykinase (GTP) [EC:4.1.1.32] |
| TCA_CS | CS, gltA; citrate synthase [EC:2.3.3.1] |
| TCA_IDH | IDH1, IDH2, icd; isocitrate dehydrogenase [EC:1.1.1.42] |
| TCA_susD | susD; starch-binding outer membrane protein, SusD/RagB family |
| Ace_pta | E2.3.1.8, pta; phosphate acetyltransferase [EC:2.3.1.8] |
| Ace_ackA | ackA; acetate kinase [EC:2.7.2.1] |
| Ace_acs | ACSS1_2, acs; acetyl-CoA synthetase [EC:6.2.1.1] |
| rTCA_porA | porA; pyruvate ferredoxin oxidoreductase alpha subunit [EC:1.2.7.1] |
| rTCA_aclA | aclA; ATP-citrate lyase alpha-subunit [EC:2.3.3.8] |
| rTCA_aclB | aclB; ATP-citrate lyase beta-subunit [EC:2.3.3.8] |
| WL_cooS | cooS, acsA; anaerobic carbon-monoxide dehydrogenase catalytic subunit [EC:1.2.7.4] |
| CBB_rbcL | rbcL; ribulose-bisphosphate carboxylase large chain [EC:4.1.1.39] |
| CBB_rbcS | rbcS; ribulose-bisphosphate carboxylase small chain [EC:4.1.1.39] |
| CBB_prkB | PRK, prkB; phosphoribulokinase [EC:2.7.1.19] |
| WL_acsB | acsB; acetyl-CoA synthase [EC:2.3.1.169] |
| WL_fhs | fhs; formate--tetrahydrofolate ligase [EC:6.3.4.3] |
| Nitrogen_amt | amt, AMT, MEP; ammonium transporter, Amt family |
| Nitrogen_gcvT | gcvT, AMT; aminomethyltransferase [EC:2.1.2.10] |
| Nitrogen_gdhA | GLUD1_2, gdhA; glutamate dehydrogenase (NAD(P)+) [EC:1.4.1.3] |
| Nitrogen_nxrA | narG, narZ, nxrA; nitrate reductase / nitrite oxidoreductase, alpha subunit [EC:1.7.5.1 1.7.99.-] |
| Nitrogen_narG | narG, narZ, nxrA; nitrate reductase / nitrite oxidoreductase, alpha subunit [EC:1.7.5.1 1.7.99.-] |
| Nitrogen_narB | narB; ferredoxin-nitrate reductase [EC:1.7.7.2] |
| Nitrogen_napA | napA; periplasmic nitrate reductase NapA [EC:1.7.99.-] |
| Nitrogen_nirB | nirB; nitrite reductase (NADH) large subunit [EC:1.7.1.15] |
| Nitrogen_nrfA | nrfA; nitrite reductase (cytochrome c-552) [EC:1.7.2.2] |
| Nitrogen_nirK | nirK; nitrite reductase (NO-forming) [EC:1.7.2.1] |
| Nitrogen_norB | norB; nitric oxide reductase subunit B [EC:1.7.2.5] |
| Nitrogen_nosZ | nosZ; nitrous-oxide reductase [EC:1.7.2.4] |
| Sulfur_soxA | soxA; L-cysteine S-thiosulfotransferase [EC:2.8.5.2] |
| Sulfur_soxB | soxB; S-sulfosulfanyl-L-cysteine sulfohydrolase [EC:3.1.6.20] |
| Sulfur_soxX | soxX; L-cysteine S-thiosulfotransferase [EC:2.8.5.2] |
| Sulfur_soxY | soxY; sulfur-oxidizing protein SoxY |
| Sulfur_soxZ | soxZ; sulfur-oxidizing protein SoxZ |
| Sulfur_fccA | fccA; cytochrome subunit of sulfide dehydrogenase |
| Sulfur_fccB | fccB; sulfide dehydrogenase [flavocytochrome c] flavoprotein chain [EC:1.8.2.3] |
| Sulfur_sat | sat, met3; sulfate adenylyltransferase [EC:2.7.7.4] |
| Sulfur_aprA | aprA; adenylylsulfate reductase, subunit A [EC:1.8.99.2] |
| Sulfur_dsrA | dsrA; dissimilatory sulfite reductase alpha subunit [EC:1.8.99.5] |
| Sulfur_phsA | phsA, psrA; thiosulfate reductase / polysulfide reductase chain A [EC:1.8.5.5] |
| Sulfur_sqr | sqr; sulfide:quinone oxidoreductase [EC:1.8.5.4] |

**Figure S1. Samples from SWIR hydrothermal vents.**


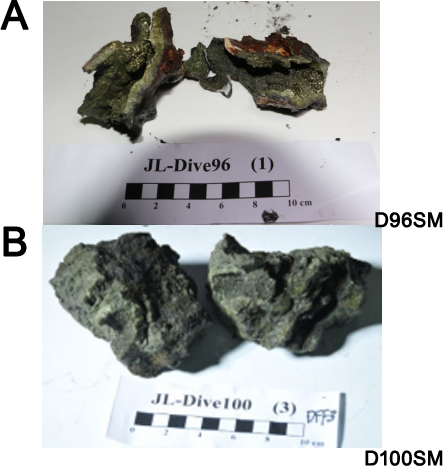


**Figure S2**. Main minerals in sulfide rocks

X-Ray Diffraction (XRD) analysis was applied to examine mineral composition of D95S (A) and D100S (B).


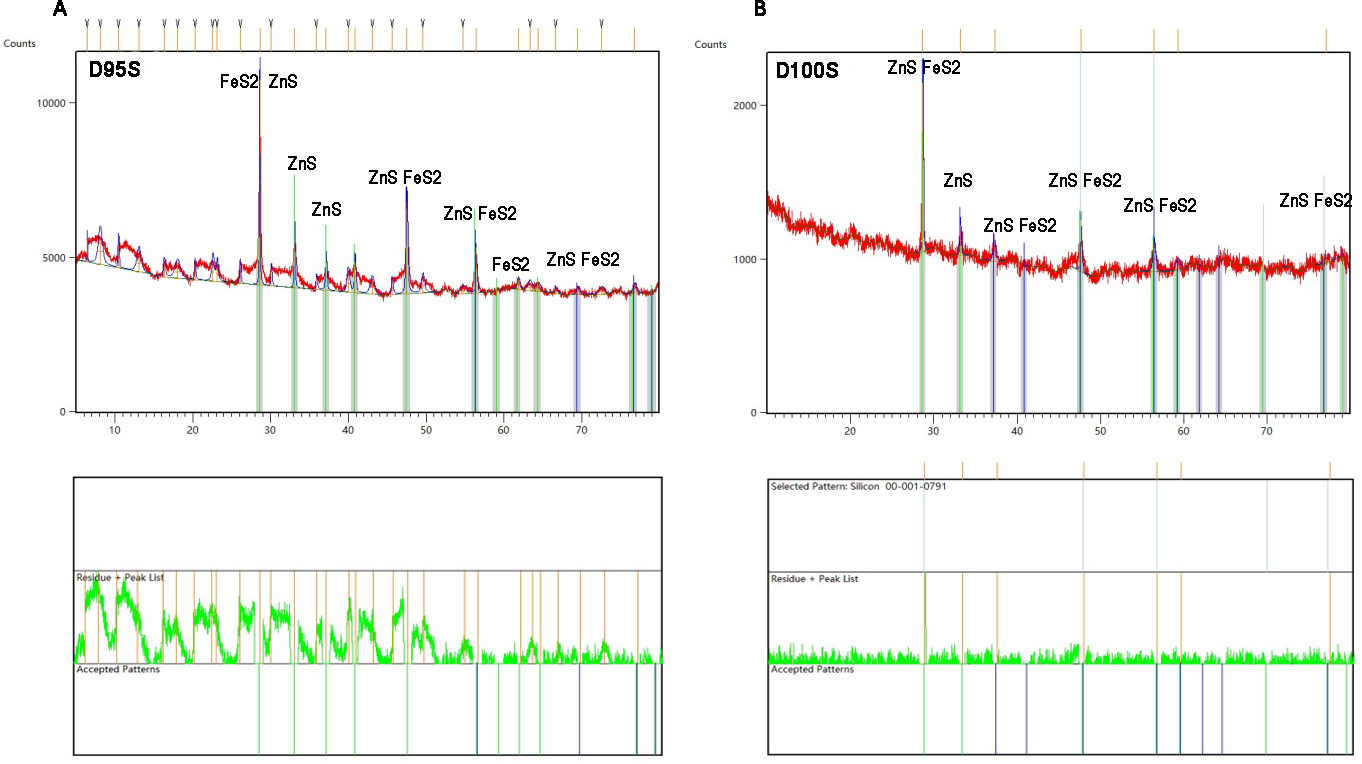

Supplement: Supplementary file 1 [file Table_1.docx]
